# A potential controlling approach on surface ozone pollution based upon power big data

**DOI:** 10.1007/s42452-022-05045-5

**Published:** 2022-05-10

**Authors:** Xin Wang, Weihua Gu, Feng Wang, Li Liu, Yu Wang, Xuemin Han, Zhouqing Xie

**Affiliations:** 1 State Grid Anhui Electric Power Research Institute, Hefei, 230026 Anhui China; 2 Department of Environmental Science and Engineering, University of Science and Technology of China, Hefei, 230026 Anhui China; 3 State Grid Anhui Electric Power CO. LTD, Hefei, 230026 Anhui China

**Keywords:** Surface ozone pollution, Generalized additive models (GAMs), Power big data, Volatile organic compounds(VOCs)

## Abstract

Surface ozone pollution has attracted extensive attention with the decreasing of haze pollution, especially in China. However, it is still difficult to efficiently control the pollution in time despite numbers of reports on mechanism of ozone pollution. Here we report a method for implementing effective control of ozone pollution through power big data. Combining the observation of surface ozone, NO_2_, meteorological parameters together with hourly electricity consumption data from volatile organic compounds (VOCs) emitting companies, a generalized additive model (GAM) is established for quantifying the influencing factors on the temporal and spatial distribution of surface ozone pollution from 2020 to 2021 in Anhui province, central China. The average R^2^ value for the modelling results of 16 cities is 0.82, indicating that the GAM model effectively captures the characteristics of ozone. The model quantifies the contribution of input variables to ozone, with both NO_2_ and industrial VOCs being the main contributors to ozone, contributing 33.72% and 21.12% to ozone formation respectively. Further analysis suggested the negative correlation between ozone and NO_2_, revealing VOCs primarily control the increase in ozone. Under scenarios controlling for a 10% and 20% reduction in electricity use in VOC-electricity sensitive industries that can be identified by power big data, ozone concentrations decreased by 9.7% and 19.1% during the pollution period. This study suggests a huge potential for controlling ozone pollution through power big data and offers specific control pathways.

**Supplementary Information:**

The online version contains supplementary material available at 10.1007/s42452-022-05045-5.

## Article Highlights

- Surface ozone pollution in central China was investigated during the prevalence of the COVID-19 (2020.1–2021.5)
- NO_2_ and industrial VOCs contributing 33.72% and 21.12% to ozone formation
- Potential controlling pathway was proposed

**Supplementary Information:**

The online version contains supplementary material available at 10.1007/s42452-022-05045-5.

## Introduction

Ozone (O_3_) pollution in the troposphere has been of great concern over the past few decades. Tropospheric O_3_ is a strong oxidant that affects atmospheric oxidation capacity [1]. It is harmful to human fitness [2, 3] and affects vegetation [4, 5]. In addition to fine particulate matter (PM2.5), tropospheric ozone is another serious air pollution issue in China [6, 7]. Since the implementation of the Clean Air Action Plan which was initiated in 2013, there has been a significant decrease in fine particulate matter (PM2.5) concentrations [8]. However, ground-level ozone pollution in China remains severe. Surface ozone pollution in China has been reported to increase since 2013 [9, 10]. There is also a global trend towards increased ozone pollution, especially in urban areas [11].

O_3_ is a typical secondary pollutant. The formation of O_3_ pollution depends on emissions of ozone precursors and local meteorological conditions. Tropospheric ozone pollution is mainly generated by atmospheric photochemical reactions of precursors (NO_x_ and VOCs) during the exposure to daylight [12]. Meteorological conditions affect ozone production by altering natural emissions and chemical rates [13]. The mechanisms of ozone formation are mainly divided into VOC-limited photochemical regime and NO_X_-limited photochemical regime [14]. Studies have shown that in China’s major urban and industrial areas, ozone production is mainly limited by VOCs due to high NO_X_ levels [15, 16]. China now has a well-established network of NOx observation sites and is also promoting control measures for NOx (control of emissions from coal-fired power plants). However, given the complexity of the VOC species, observations of VOCs are difficult to obtain, and there is a lack of corresponding data to support the control pathways for VOCs.

Considering the complex non-linear response between ozone and precursors and meteorological factors, chemical tracer models (CTMs) are commonly used to estimate tropospheric ozone [17–19]. These models are often complex, require significant computational resources and are dependent on the updating of emission inventories. In recent years, statistical models, including machine learning models, have been used in ozone pollution studies. As a common statistical model in the environmental field, GAMs have no prior assumptions between variables and the results of GAMs are more interpretable than machine learning models. GAMs have been used to analyze the relationship between ozone pollution and meteorological conditions [20, 21]. However, few studies have considered the effect of precursors in GAM modelling due to the lack of observational data on VOCs.

Industrial sources of VOCs are the most important source of non-methane VOCs in Chinese urban areas [22]. For VOC emitting enterprises, the electricity consumption data directly reflects the production and operation status of these enterprises and also contains information on the pollutant emission status of these enterprises. Electricity consumption is closely related to pollutant emissions and carbon emissions [23–25], but few studies apply electricity data to air pollution analysis. Therefore, high temporal resolution electricity consumption data from VOC emitting companies may be a valid indicator of VOC emissions from industrial sources. At the same time, the electricity data is more conducive to the government’s implementation of precise control over the relevant key emission industries.

This study quantitatively investigated the influencing factors of ozone pollution based on GAMs in Anhui Province, China together with high resolution electricity consumption data. The result provides insight to understand the change in ozone pollution and how to precisely control ozone based on electricity data to reduce VOC emissions. In the next section, we describe the sources of the data, the methods used to implement the model and the quality control. In Sect. 3, we first present the spatial and temporal distribution characteristics of ozone in Anhui Province. We then discuss the influencing factors of ozone pollution based on the GAM model and propose a phenomenological pathway to control ozone based on power big data. The major findings are summarized in Sect. 4.

## Data and methods

### Data sources

This study provides an analysis of the characteristics and driving factors of O_3_ pollution in Anhui Province from January 2020 to May 2021 (electricity consumption data is only available as early as January 2020). The near-surface pollutant data (O_3_, NO_2_) at hourly resolution were obtained from the Department of Ecology and Environment (http://sthjt.ah.gov.cn/site/tpl/5371) [26]. It should be noted that to analyze the historical trends in the spatial and temporal distribution of ozone in Anhui Province, we also extracted ozone data from state-controlled sites in Anhui Province from 2018 to 2020. Meteorological data corresponding to pollutant data, including temperature (T), relative humidity (RH), wind speed (WS), and wind direction (WD), were obtained from the NOAA website (http://www.cdc.noaa.gov) [27]. A list of major VOC-emitting enterprises and their electricity consumption data in Anhui Province were provided by the State Grid Anhui Electric Power Corporation. We selected major VOC-emitting enterprises based on previous work and the actual situation in Anhui Province, and divided them into 14 categories [28, 29]. All data were averaged hourly and subjected to strict quality control to ensure integrity and representativeness. In this time period 145,656 valid data were finally obtained.

### Generalized additive model method

In this study, we used GAMs to analyze the main factors affecting the variation in ozone concentration in the Anhui Province. Generalized additive model is a non-linear 1regression model, a semi-parametric extension of the generalized linear model (GLM) that can directly deal with the complex non-linear relationships between response variables and multiple explanatory variables [30, 31]. The model construction is based on the gam function with “mgcv” package in R software [32, 33]. “mgcv” is an R package for estimating GAMs. The equation is as follows:

$$ {\text{g}}\left( \upmu \right) = f_{1} \left( {{\text{x}}_{{1}} } \right){ + }f_{2} \left( {{\text{x}}_{{2}} } \right){ + } \ldots { + }f_{k} \left( {{\text{x}}_{{\text{i}}} } \right){ + }\upvarepsilon $$

where $\mu$ represents the predicted value for the independent variables, that is, ozone concentration; $x_{i} \left( {i = 1,2, \ldots ,n} \right)$ are predictors (e.g., WS, WD, T, RH, ELE, etc.); $f_{k} ()$ is smooth functions of the predictors; ${\text{g}}\left( \upmu \right)$ is the link function; ${\upvarepsilon }$ is intercept. Gong, et al. [34] proposed that the distribution of ozone is close to Gaussian distribution and identity link is suitable for ozone in GAM. Therefore we used Gaussian distributions and the identity link function in our research, which means ${\text{g}}\left( \upmu \right) = \upmu$. Penalized cubic regression splines (CRS) were used to smooth the function to ensure that the model was not over-fitted or under-fitted [35].

### Model parameter selection and quality control

To ensure the validity of the GAM model input predictors, we determined the input predictors of the model based on the Akaike information criterion (AIC) and R^2^ [36]. When a valid predictor is added to the model, the AIC value should decrease while R^2^ increases [34]. Based on the above criteria, we tested the 20 variables selected by adding them to the model one by one. The variables included one air pollutant variable (NO_2_), five meteorological variables, and 14 electricity consumption variables. A description of the variables used in our study is presented in Table S1 (Online Resource 1). Fig. S1 (Online Resource 1) shows how the AIC and R^2^ values change as the variables increase in the modeling of Hefei. The AIC value decreased monotonically with increasing variables, and R^2^ increased monotonically with increasing variables. This indicates that the model does not appear to be over-fitted while improving the goodness of fit.

We evaluated the performance of the model using the gam.check function in the mgcv package. Fig. S2 (Online Resource 1) illustrates the model quality control results for Hefei City. The residuals conform to a normal distribution and show a random distribution with no significant trend. The fitted and observed values of ozone were well matched after the fit.

## Results and discussion

### Spatial and temporal distribution characteristics

**Fig. 1:**
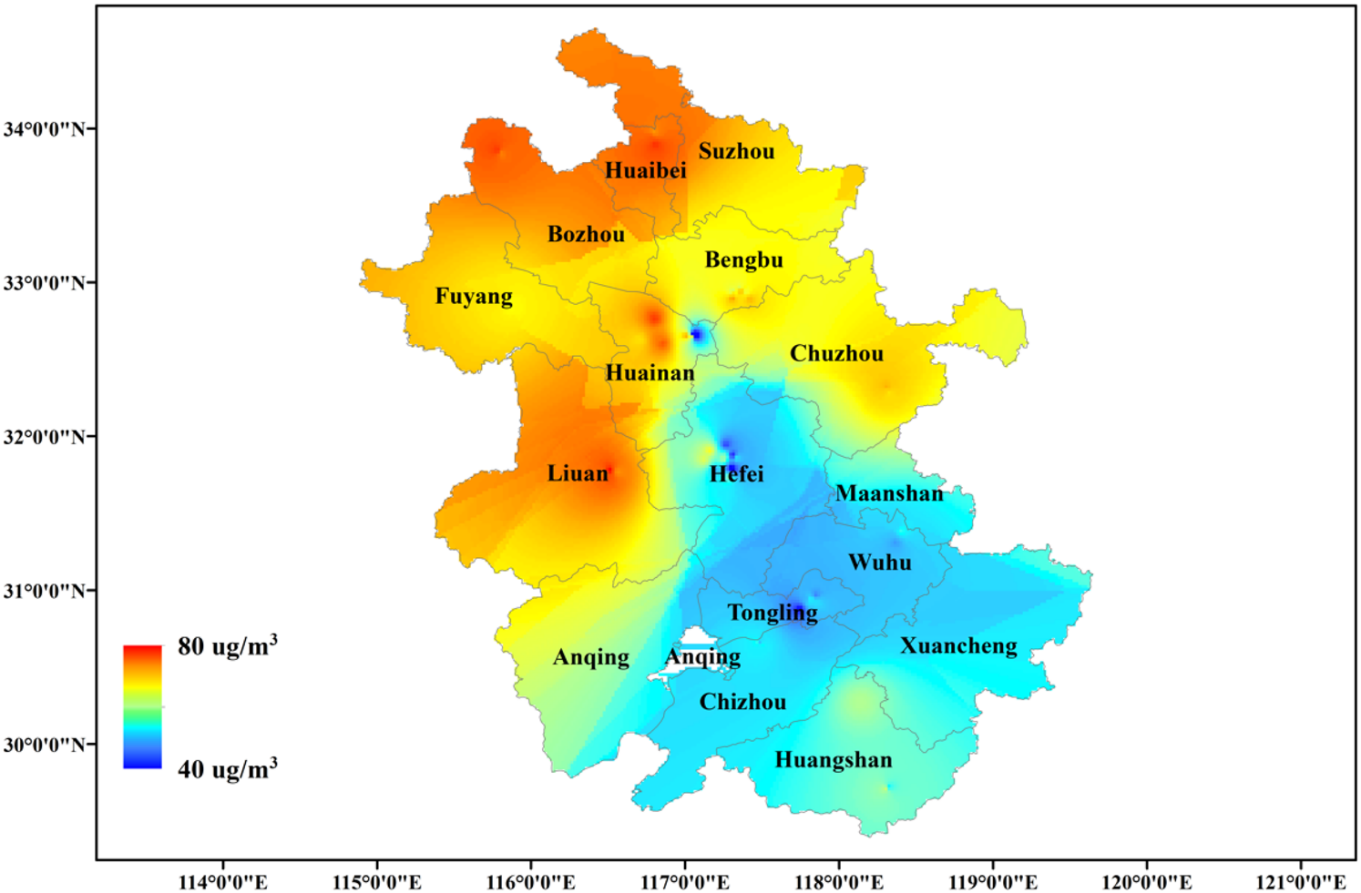
Spatial distribution characteristics of O_3_ concentrations in Anhui Province in 2020. The colours represent the annual average of ozone concentrations

Anhui Province, an important province in East China in terms of population, economy, transportation, and agriculture, has faced serious air pollution problems in recent years. Figure 1 shows the spatial distribution characteristics of the annual mean O_3_ concentrations in the Anhui Province in 2020. The overall spatial distribution of O_3_ concentrations in Anhui Province shows a clear distribution trend of higher concentrations in the north than in the south. The relatively dry climate in northern Anhui (mainly north of the Huai River) is more conducive to the formation and accumulation of O_3_.

**Fig. 2:**
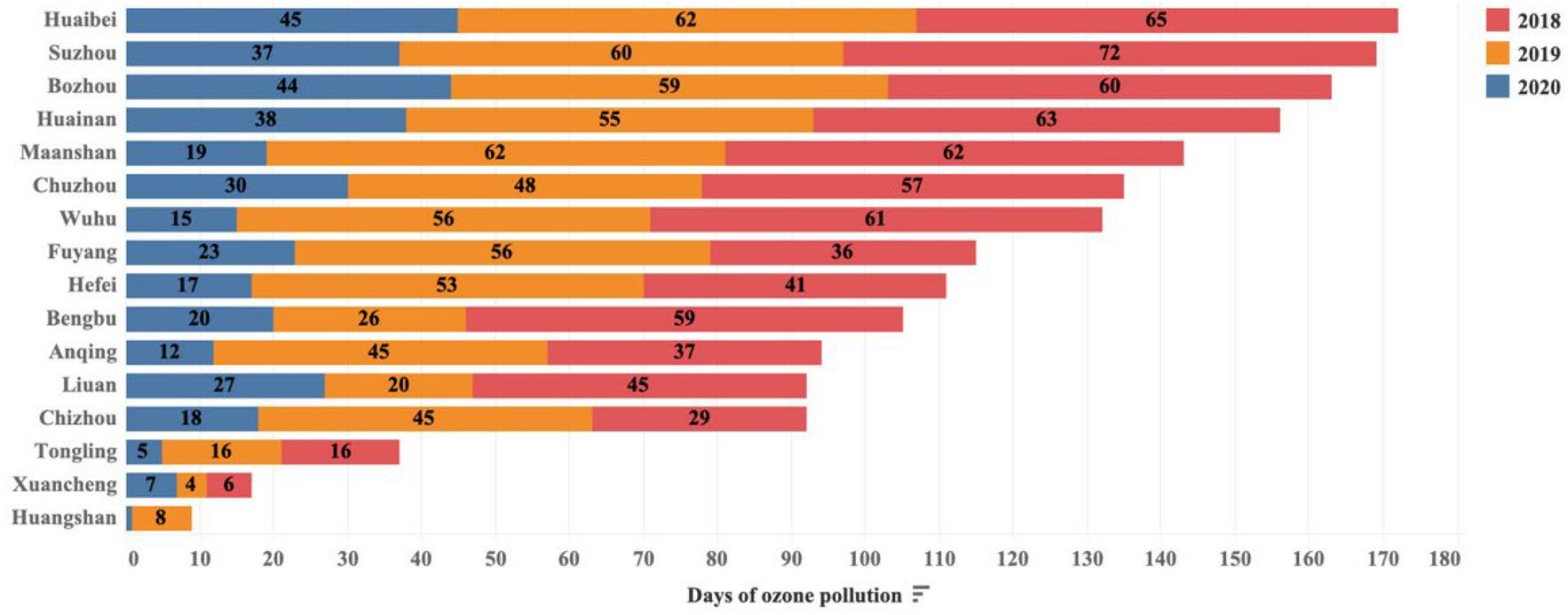
Days of ozone pollution in 16 cities in Anhui Province, 2018–2020. Here ozone pollution is defined as a maximum daily 8-h O_3_ mass concentration of more than 160 μg/m^3^

Exceedances of ozone concentrations in Anhui province were shown in Fig. 2, based on the air quality reference standard issued by the World Health Organization (ozone pollution is defined as a maximum daily 8-h O_3_ mass concentration of more than 160 μg/m^3^ [37]. All cities in Anhui Province face different levels of ozone pollution. There was a notable decrease in the number of ozone pollution days in 2020 (n = 358) relative to 2019 (n = 675) and 2018 (n = 709) (Online Resource 1, Table S2). Decrease in ozone concentrations in 2020 relative to the previous two years is closely related to the nationwide shutdown of production in China due to COVID-19. It was reported that ozone concentrations increased in some cities (17% in Europe, 36% in Wuhan) during the COVID-19 lockdown in the presence of reduced precursor emissions [38], which is contrary to the decline observed in Anhui. The variation in ozone concentrations between cities during the COVID-19 shutdown is controlled by the severity of the shutdown measures, the city’s own emissions and differences in meteorological conditions. In VOC-limited cities, ozone concentrations may increase if the emissions reduction from the shutdown only affects NO_X_ and VOC emissions are not reduced. Whereas, if both precursors are reduced together, then ozone concentrations may fall. Therefore, ozone pollution needs to be discussed in separate modelling for different cities.

### Quantify the effect of variables on ozone based upon GAM modeling

**Fig. 3:**
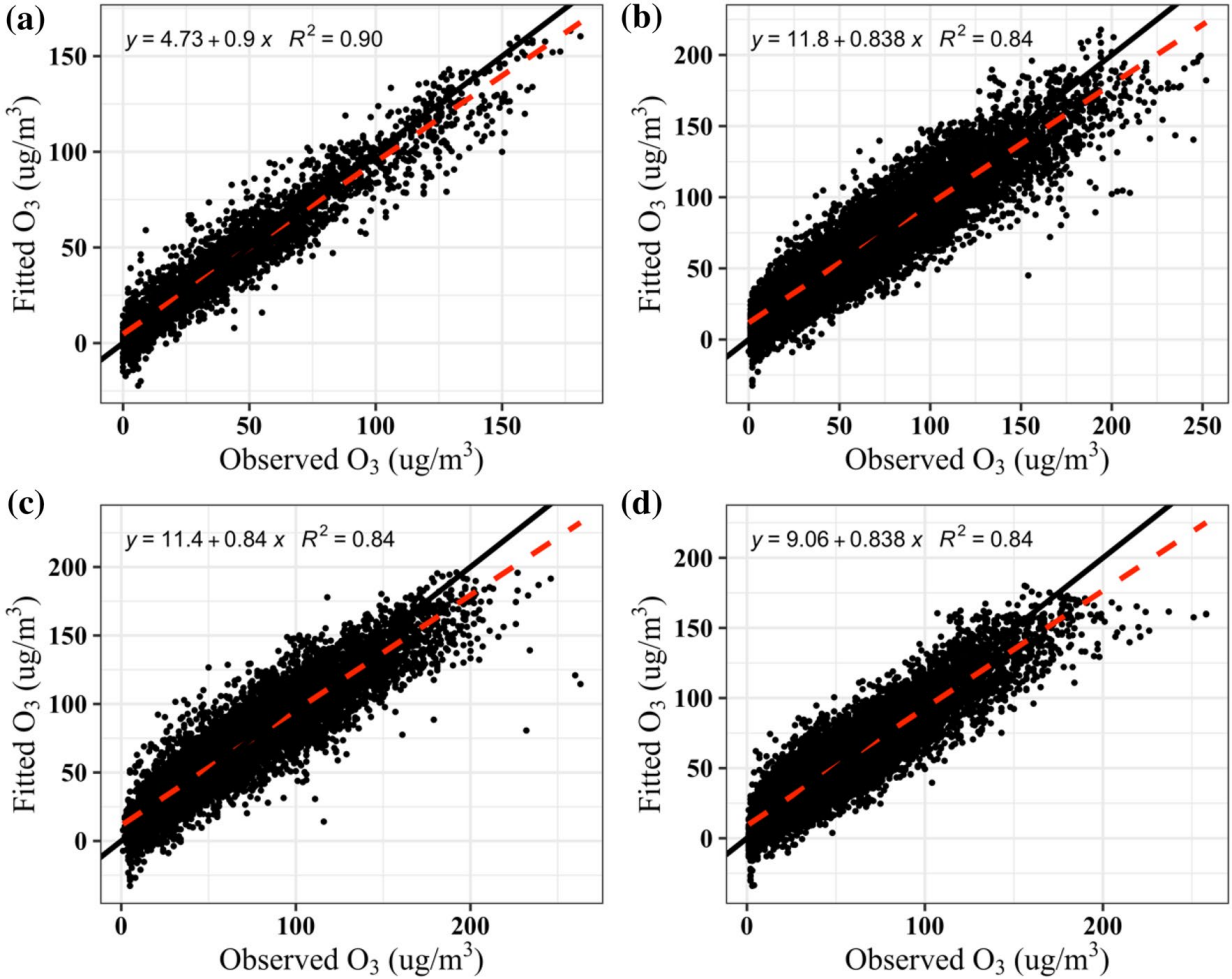
Relationships between the observed and the fitted ozone concentrations in Chizhou (**a**), Huaibei (**b**), Suzhou (**c**) and Hefei (**d**)

GAM modeling of the 20 variables listed in Table S1 (Online Resource 1) was used to fit the hourly resolution ozone concentrations in Anhui. The spatial resolution of our model predictions is for integrated city surface areas. Table 1 shows the results of modeling 16 cities in Anhui Province, characterizing the goodness of fit by adjusted R^2^ and root mean square error (RMSE). The R^2^ value ranges from 0.76 (Huangshan) to 0.90 (Chizhou), with an average of 0.82. RMSE ranges from 18.63 (Bozhou) to 10.75 μg/m^3^ (Bozhou), with an average of 16.19 μg/m^3^. We also calculated the normalized root mean square error (NRMSE, RMSE divided by the mean of the observations), with an average of 0.25. Figure 3 shows the relationships between the observed and the fitted ozone concentrations in four cities in Anhui. In general, a GAM model is considered to have a good interpretation of the response variable when the adjusted value of R^2^ exceeds 0.5 [39]. In comparison with previous studies [21, 35, 39], we believe that GAM models can capture the characteristics of ozone and the results are reliable.

**Table 1:** Summary of the performance of GAMs in Anhui

City	Adjusted R^2^	Absolute residuals (μg/m^3^)	RMSE (μg/m^3^)	NRMSE
Bozhou	0.81	13.98	18.63	0.25
Luan	0.82	12.96	16.73	0.22
Hefei	0.84	12.43	16.03	0.29
Anqing	0.83	11.83	15.12	0.22
Xuancheng	0.79	11.44	14.89	0.22
Suzhou	0.84	13.13	17.28	0.24
Chizhou	0.9	8.07	10.75	0.23
Huaibei	0.84	13.56	17.67	0.24
Huainan	0.83	13.14	16.92	0.23
Chuzhou	0.8	14.0	18.42	0.27
Wuhu	0.8	13.24	17.62	0.32
Bengbu	0.82	12.41	15.88	0.23
Tongling	0.83	10.02	13.0	0.24
Fuyang	0.83	12.84	16.43	0.24
Maanshan	0.81	13.76	17.92	0.3
Huangshan	0.76	12.29	15.77	0.25

The results of F test for GAM reflect the variance contribution of each predictor variable to the response variable [40]. The F value of a single independent variable divided by the sum of the F values of all the independent variables can represent the contribution of this variable to the change in the predictor variable [35]. We calculated the relative contribution of each variable based on the F test, and the results are listed in Table 2. NO_2_ is one of the most important O_3_ precursors. Its contribution to ozone change in Anhui Province was 33.72% in average, which was the highest of all variables. The electricity consumption parameter (sum of 14 industries) refers to industrial sources of VOC emissions, accounting for 21.12% in average. The total contribution of NO_2_ and electricity parameters amounted to 54.84%, which emphasizes the dominance of precursors in the ozone generation process. As emissions from other VOC sources (e.g. solvent use, natural sources) were not considered in this study, the actual VOC contribution may be higher. Among the meteorological parameters, temperature and humidity are the most important parameters affecting ozone variability. In this study, the two contributions of the most important meteorological parameters, relative humidity and temperature to ozone were 12.23% and 9.00%, respectively. Hu et al. [21] found that temperature and humidity may be the most significant meteorological factors influencing ozone concentrations in Chinese cities, which is consistent with our findings. Wind direction and speed, which indicate ozone transport and removal, account for a relatively lower contribution of 2.63% and 1.12%, respectively. This may be because transport and removal effects are important during specific ozone pollution events; therefore, they do not contribute much on a year-round scale.

**Table 2:** Relative importance of variables in 16 cities, including temperature (T), relative humidity (RH), wind speed (WS), wind direction (WD), hour of day (HOD), concentration of NO_2_ (NO_2_) and electricity consumption (ELE)

City	s(HOD) (%)	s(NO2) (%)	s(RH) (%)	s(T) (%)	s(WD) (%)	s(WS) (%)	s(ELE) (%)
Anqing	19.16	41.60	11.75	3.90	3.03	1.56	19.00
Bengbu	18.13	35.47	12.17	10.14	4.30	0.73	19.07
Bozhou	22.33	26.52	10.94	14.78	2.13	0.70	22.60
Chizhou	17.26	39.43	12.51	8.00	2.87	0.89	19.04
Chuzhou	18.68	36.86	12.25	6.44	3.29	0.93	21.55
Fuyang	19.16	36.15	14.17	7.32	1.78	0.97	20.46
Hefei	19.84	26.98	8.23	15.96	3.83	1.15	24.01
Huaibei	17.34	35.22	10.49	13.88	2.59	0.63	19.85
Huainan	20.03	22.00	9.35	15.27	2.84	1.23	29.27
Huangshan	33.09	17.83	24.88	1.51	4.60	3.56	14.54
Luan	15.38	38.75	10.09	6.02	1.94	0.64	27.18
Maanshan	21.88	37.88	11.33	10.70	2.63	0.85	14.73
Suzhou	21.30	41.40	6.42	10.40	1.22	0.86	18.41
Tongling	16.60	46.20	6.87	7.80	1.21	1.62	19.69
Wuhu	21.34	36.43	11.24	8.76	2.21	0.50	19.53
Xuancheng	21.53	20.80	22.91	3.06	1.62	1.14	28.94
Mean	20.19	33.72	12.23	9.00	2.63	1.12	21.12
Std	3.87	8.04	4.85	4.25	0.98	0.70	4.23

#### NO_2_

**Fig. 4:**
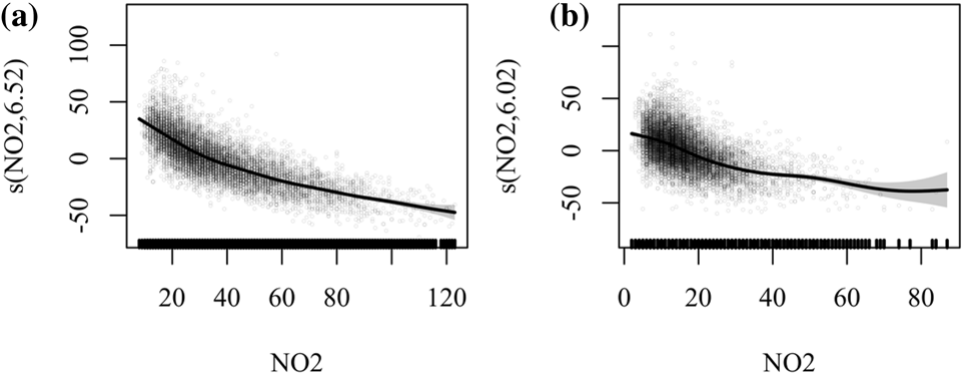
Impacts of NO_2_ on O_3_ in GAM in Tongling (**a**) and Huangshan (**b**). The Y-axis in each subplot represents the smoothing function term for each predictor, and the numbers in brackets represent degrees of freedom. Black markers on the X-axis represent the distribution of the predictors (corresponding to the scatter points in the plot)

In our GAMs modeling results, O_3_ was negatively correlated with NO_2_ in all 16 cities in Anhui. Figure 4 shows the relationship between O_3_ and NO_2_ in Tongling and Huangshan, both showing an evident negative correlation. Schroeder, et al. [41] proposed that when VOC concentrations are stable, the relationship between O_3_ and NO_2_ driven by nonlinear O_3_-NO_x_ chemistry follows a log-normal distribution. For high NO_x_ concentrations (NO_x_ excess zone), O_3_ concentrations increase with decreasing NO_x_ concentrations, while when NOx concentrations are low (NO_x_ sensitive zone), O_3_ concentrations change in line with NO_x_. This indicates that Anhui Province is still in a NO_x_ excess zone, and O_3_ pollution is mainly controlled by VOCs.

Thus, although the contribution of NO_2_ was the highest of all the variables, it was negatively correlated with ozone. This means that the control of NO_2_ may exacerbate ozone pollution to some extent. This means that if VOC control is neglected, control of NO2 may have exacerbated ozone pollution to some extent. Sicard, et al. [38] reported that the lockdown during COVID-19 caused a substantial decrease in NO_x_ (~ 56%) and an increase in O_3_ (~ 36%) in Wuhan. Chen, et al. [42] compared ozone pollution in China with ozone pollution in the USA in the 1990s and concluded that stricter NOx controls can improve O_3_ pollutions over industrialized areas. In the abatement scenario of COVID-19, the Anhui region has not reached a NOx sensitive area. Our results may imply that a greater emphasis on reducing anthropogenic VOCs may be a more effective pathway for ozone control in most industrial areas until there are greater improvements in NOx concentrations.

#### Electricity consumption

**Fig. 5:**
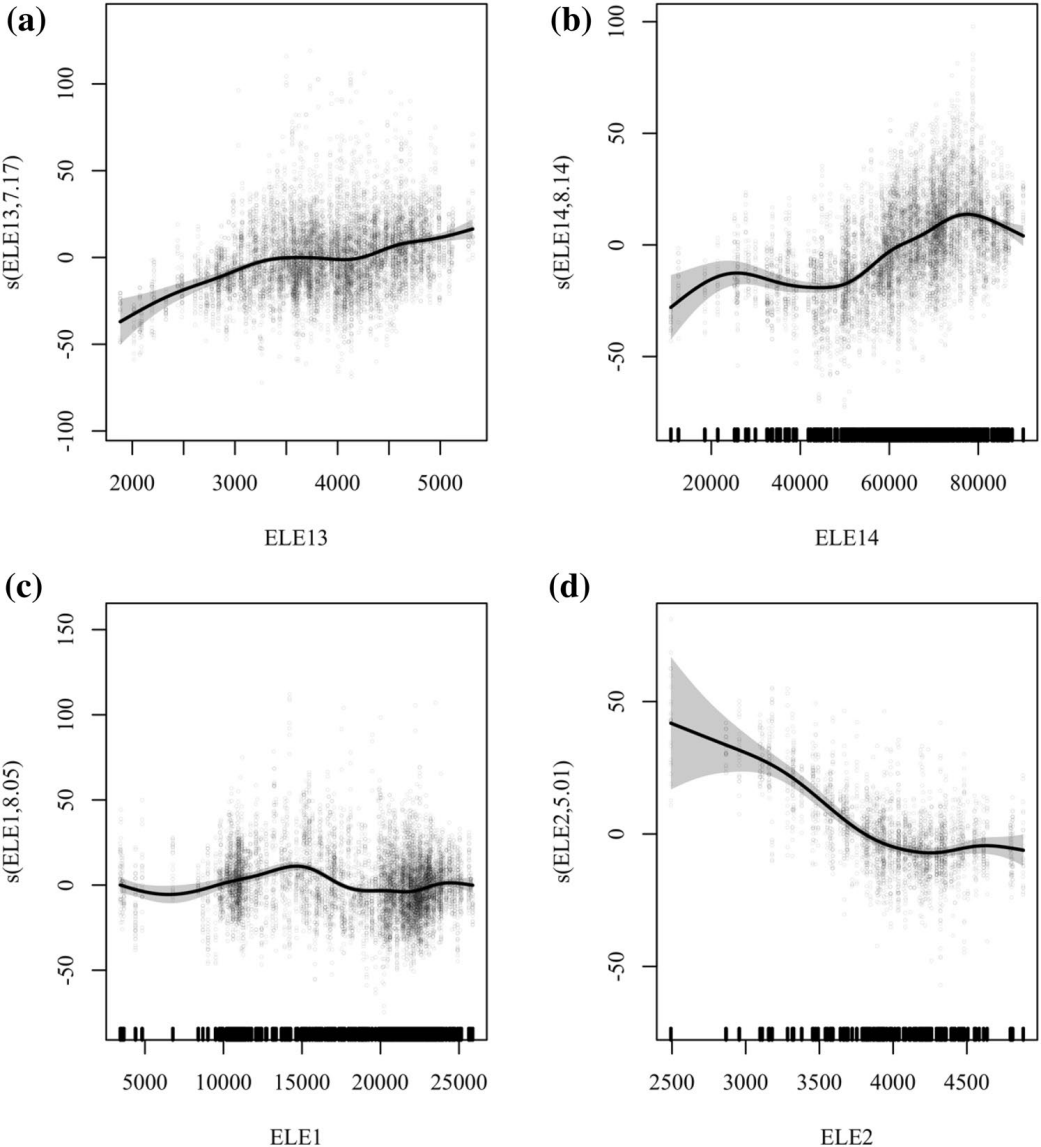
Plot of partial dependence of electricity consumption variables on O_3_ in representative cities. Panel **a** shows the relationship between O_3_ and ELE13 in Bozhou; Panel **b** shows the relationship between O_3_ and ELE14 in Fuyang; Panel **c** shows the relationship between O_3_ and ELE1 in Huaibei; Panel **d** shows the relationship between O_3_ and ELE2 in Chizhou

As the main VOC emitting industries vary from city to city, the production and electricity consumption patterns of each industry also vary considerably. After analyzing the partial dependence diagrams (Fig. 5) between electricity and ozone for all industries, we divided these industries into 2 categories.

The first category of industries we refer to as the VOC-electricity sensitive category. The non-metallic mineral products industry in Bozhou and the metal products industry in Fuyang, illustrated in Fig. 5a and b, are representative of this. The distinctive feature of these industries is that the increase in electricity consumption contributes to the increase in local ozone concentrations. The VOC emissions from this type of industry may be closely related to their production and electricity consumption processes. For such enterprises, we can effectively control them by limiting electricity use, as their increased electricity use contributes to local ozone formation.

The second group of industries we call the VOC-electricity insensitive category. Chemical raw material and chemical product manufacturing in Huaibei and chemical fibre manufacturing in Chizhou, illustrated in Fig. 5c and d, fall into this category. This category is characterised by the fact that there is no tendency for ozone to increase with electricity consumption. The VOC emissions from these enterprises usually involve the use of solvents. Therefore, the VOC emission phase in this category may be concentrated before or after the production activity, while during the production activity there is less direct emission of VOC. For this type of enterprises, the environmental authorities have to control their emissions according to the actual situation and not simply through the use of electricity.

#### Meteorology

**Fig. 6:**
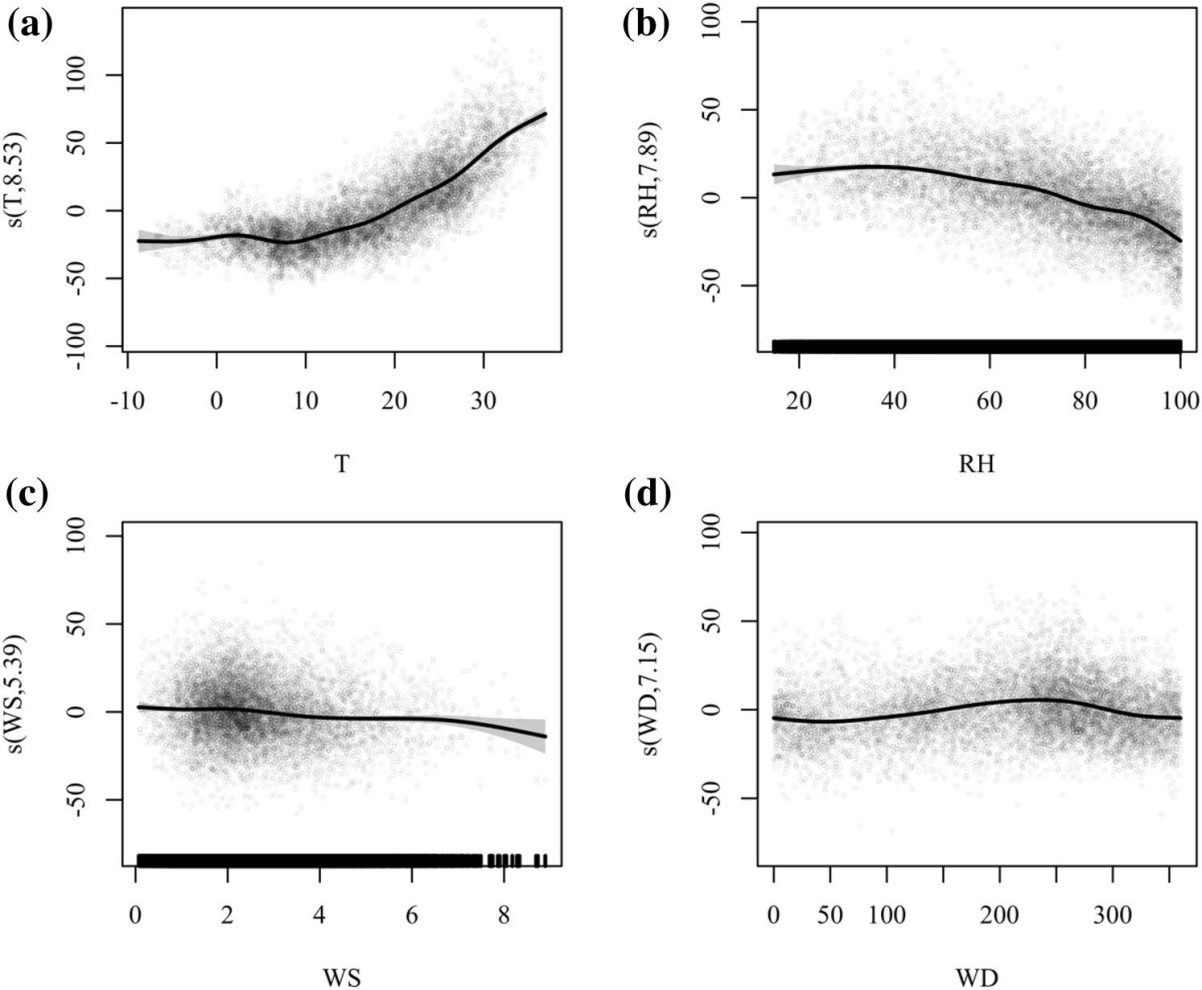
Plot of partial dependence of meteorological variables on O_3_ in representative cities. Panel **a** show the relationship between O_3_ and T in Hefei; Panel **b** show the relationship between O_3_ and RH in Huangshan; Panel **c** show the relationship between O_3_ and WS in Huangshan; Panel **d** shows the relationship between O_3_ and WD in Hefei

Many studies have been conducted on the relationship between ozone and meteorological parameters. According to previous studies, ozone pollution is prone to occur on days with strong sunlight and low wind speeds [14]. Our modeling results show that ozone concentrations in Anhui Province increase with increasing T (Fig. 6a) and decrease with increasing RH (Fig. 6b), which is consistent with previous research findings [20, 21].

Higher wind speeds tend to promote ozone removal, but most of Anhui cities in the model do not exhibit this feature, except for Huangshan. The relative contribution of WD and WS in Huangshan was the highest of all cities, at 4.60% and 3.56%, respectively. As shown in Fig. 6c, Huangshan City shows a decreasing trend of ozone under high wind speed conditions, which may be strongly related to the topography of Huangshan City. The city of Huangshan has a vast mountainous landscape, with the highest elevation of Huangshan reaching 1864 m. Valley winds have a strong effect on pollutants. Highland winds transport O_3_ and other pollutants to the mountains during the day, while valley winds send them back to plain areas at night [43, 44]. Figure 6d illustrates the relationship between ozone and wind direction in Bengbu City. As can be seen from the figure, ozone concentrations in Bengbu City reach their highest around a wind direction of 250°. This could mean that Bengbu is more susceptible to other cities in northern Anhui, such as Huainan and Bozhou.

The hour of day (HOD) predictor mainly indicates the daily ozone variation characteristics. The daily ozone variation is primarily influenced by a combination of solar radiation and meteorological parameters such as temperature and wind speed. Huangshan City has the highest relative contribution of HOD to ozone among all the cities. The pattern of daily ozone variation in 16 cities in Anhui Province is very similar, with concentrations starting to rise during the day from approximately 7 to 8 am, reaching a cumulative maximum ozone concentration at approximately 6 pm, and then starting to fall. Because Huangshan City is the least ozone-polluting city in Anhui Province, ozone levels may be more controlled by natural sources and meteorological conditions.

### Potential controlling pathway of ozone pollution

**Fig. 7:**
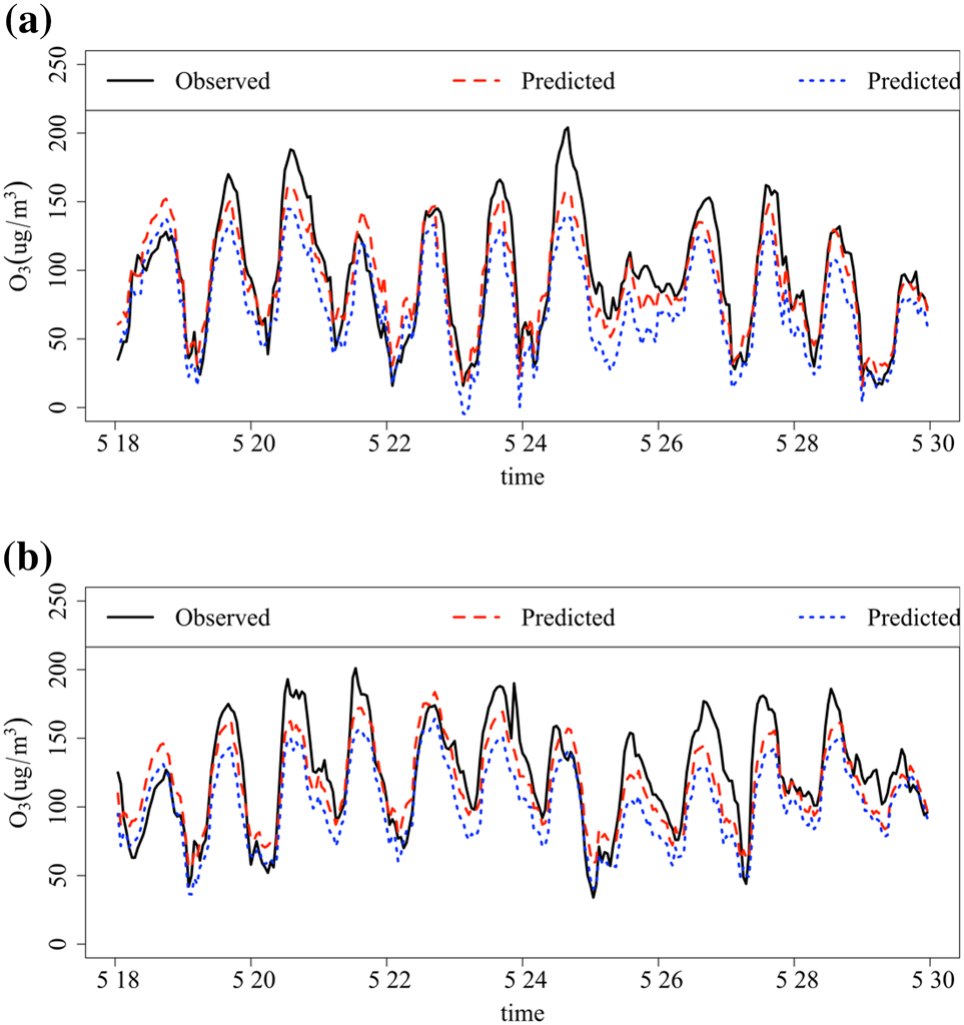
Actual observed and model-predicted concentrations of ozone in Anqing (**a**) and Fuyang (**b**) during pollution events. The red dashed line represents the prediction using the original data. The blue dashed line represents the prediction for the scenario with a 50% decrease in the ELE14

Based on the ozone control approach presented in Sect. 3.2.2, we first selected individual industries for the abatement sensitivity experiments. We selected an ozone pollution event from 18 May 18, 2020, to 3 May 30, 2020. As shown in Fig. 7, the ozone scenarios simulated by the model for Anqing and Fuyang cities with a 50% reduction in electricity consumption for ELE14 (non-metallic mineral products industry electricity consumption) are shown. Ozone concentrations decreased by 18.8% and 12.6% in Anqing City and Fuyang City, respectively, throughout the pollution event period. It can be seen that targeted control of individual key industries can achieve considerable results.

**Fig. 8:**
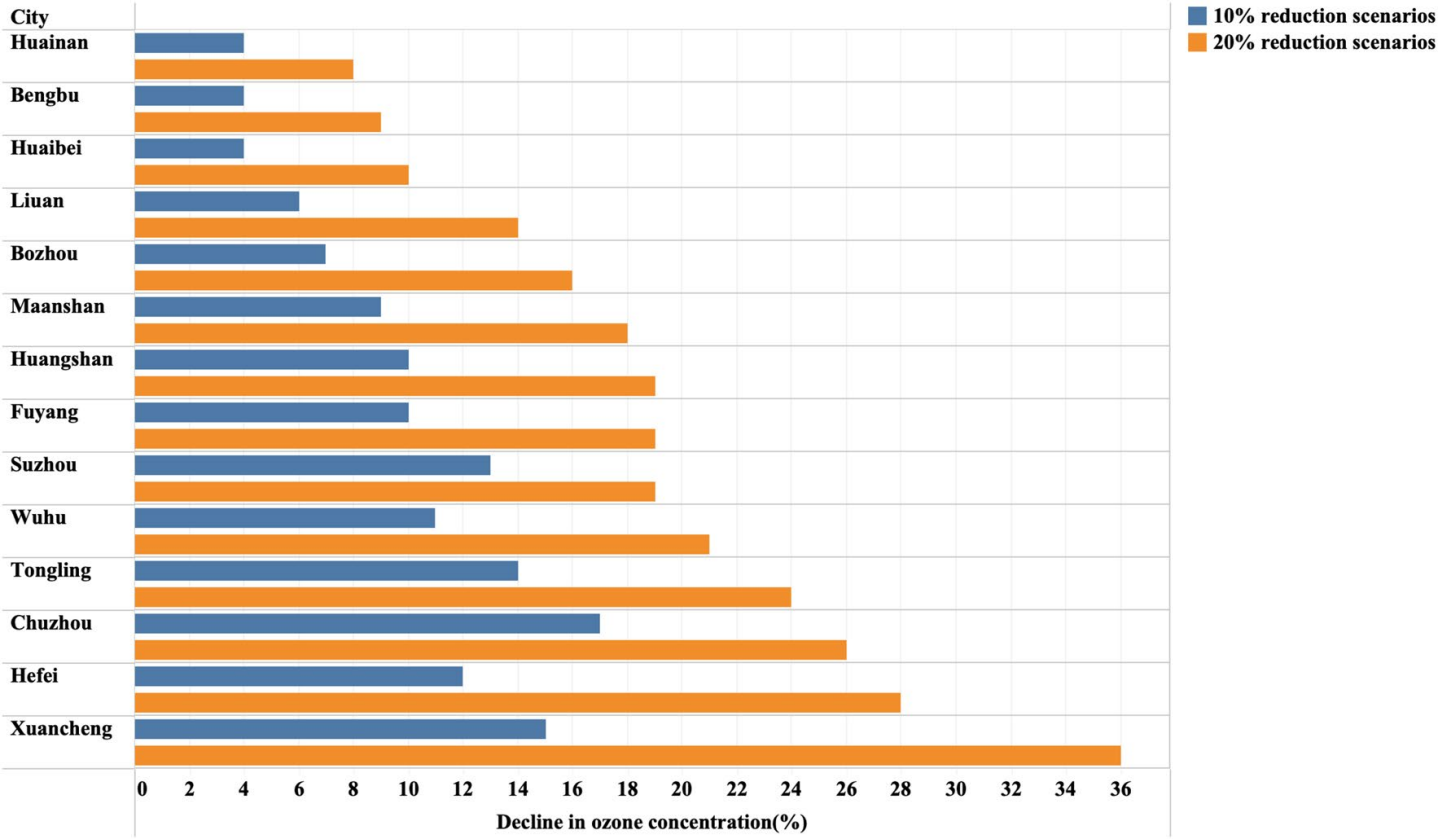
The effect of ozone pollution control under the scenarios of 10% and 20% reduction in electricity consumption for all VOC-electricity sensitive industries in Anhui

In addition to extreme reduction scenarios for individual industries, a more general approach should be reducing emissions of all VOC-limited industries. Therefore, we evaluated the effect of ozone pollution control under the scenarios of 10% and 20% reduction in electricity consumption for all VOC-electricity sensitive industries. As shown in Fig. 8, the 10% abatement scenario resulted in an average decrease in ozone concentrations of 9.7% (4–17%) during the pollution period (ozone concentrations above 160 μg/m^3^). In contrast, the 20% reduction scenario resulted in an average decrease in ozone concentrations of 19.1% (8–36%). The sensitivity of different cities to emissions reductions varies considerably. Cities with a low industrial presence are relatively insensitive to emissions reductions (Huangshan), while cities with a high industrial presence are more sensitive to emissions reductions (Hefei, Wuhu). It can be seen that there is a very large potential to reduce ozone pollution by restricting VOC emitting companies. Based on our model results, we can implement more targeted controls on ozone pollution in each municipality. During high ozone pollution seasons and in cities where ozone pollution is severe, measures to restrict electricity consumption of key local industries can be more effective in controlling local ozone pollution.

## Conclusion

This study analyzed the spatial and temporal distribution characteristics of ozone concentrations in the Anhui Province since 2018. Ozone concentrations in Anhui show a spatial pattern of higher concentrations in the north than in the south. Ozone concentrations show a significant decrease in Anhui in 2020 relative to the previous two years due to COVID-19. In contrast, ozone concentrations increased in other cities with large production shutdowns during the 2020 epidemic (e.g., Wuhan). As the industry recovers, the ozone concentrations in Anhui rebound in 2021 relative to 2020. This suggests that in Anhui Province, the control of anthropogenic precursors of ozone effectively controls ozone pollution.

Therefore, we used the electricity consumption of key emitters to represent the VOC emissions of enterprises, and based on GAMs modelling, we analyzed the main influencing factors of ozone in 16 cities in Anhui Province. The results of the model R^2^ (0.82), RMSE (16.19 μg/m^3^), and NRMSE (0.25) showed that GAMs could capture the ozone variability characteristics in Anhui Province. Among the meteorological factors, temperature and humidity are the most important factors affecting ozone variability.

The relative contribution of NO_2_ concentration was the highest of all factors. However, the relationship between NO_2_ concentration and ozone was negative. This implies that Anhui Province may still be in the VOC control area and that control measures for ozone should focus more on reducing the levels of VOCs from anthropogenic sources.

We further analyze the relationship between enterprise electricity consumption data and O_3_ for 14 industries and categorized them into VOC-electricity sensitive category and VOC-electricity insensitive category. Emission reductions for just one VOC-electricity sensitive industry have the potential to reduce ozone concentrations by more than 10% during the pollution period. Under scenarios controlling for a 10% and 20% reduction in electricity use in VOC-sensitive industries, ozone concentrations decreased by 9.7% and 19.1% during the pollution period. Therefore, we believe that controlling VOC emissions from industrial sources has great potential for ozone reduction. GAM model based on power big data may be an effective way to achieve phenomenological ozone control.

## Supplementary Information

Below is the link to the electronic supplementary material.

Supplementary file1 (DOCX 324 kb)

## Data Availability

The datasets generated during and/or analyzed during the current study are available from the corresponding author on reasonable request.
