# Supplementary material for: A potential controlling approach on surface ozone pollution based upon power big data
Source: SN Appl Sci. 2022 May 10;4(6):164. doi: 10.1007/s42452-022-05045-5 (PMC9086420; doi:10.1007/s42452-022-05045-5)
Supplement: Supplementary file 1 — Supplementary file1 (DOCX 324 kb) [file 42452_2022_5045_MOESM1_ESM.docx]

*Supplementary Information for*

**A potential controlling approach on surface ozone pollution based upon power big data**

Xin Wang^1^; Weihua Gu^2*^; Feng Wang ^3^; Li Liu ^3^; Yu Wang ^1^; Xuemin Han^1^; Zhouqing Xie^2^

^1^State Grid Anhui Electric Power Research Institute, Hefei, Anhui 230026, China

^2^Department of Environmental Science and Technology, University of Science and Technology of China, Hefei, Anhui 230026, China

^3^State Grid Anhui Electric Power CO. LTD, Hefei, Anhui 230026, China

^*^To whom correspondence should be addressed. E-mail: [huazai@mail.ustc.edu.cn](mailto:huazai@mail.ustc.edu.cn) (Weihua Gu)

**Tables**

Table S1 Variables used in this study

| **NO.** | **Variables** | **Description** | **Data source *** |
| --- | --- | --- | --- |
| **1** | **T** | **Temperature** | **3** |
| **2** | **RH** | **Relative humidity** | **3** |
| **3** | **WD** | **Wind direction** | **3** |
| **4** | **WS** | **Wind speed** | **3** |
| **5** | **NO2** | **Concentration of NO_2_** | **2** |
| **6** | **ELE1** | **Chemical raw material and chemical product manufacturing electricity consumption** | **4** |
| **7** | **ELE2** | **Chemical fibre manufacturing electricity consumption** | **4** |
| **8** | **ELE3** | **Pharmaceutical manufacturing electricity consumption** | **4** |
| **9** | **ELE4** | **Printing and recording media reproduction industry electricity consumption** | **4** |
| **10** | **ELE5** | **Furniture manufacturing electricity consumption** | **4** |
| **11** | **ELE6** | **Rubber and plastic products industry electricity consumption** | **4** |
| **12** | **ELE7** | **Automotive manufacturing electricity consumption** | **4** |
| **13** | **ELE8** | **Leather, fur, feather and feather products and footwear electricity consumption** | **4** |
| **14** | **ELE9** | **Oil, coal and other fuel processing industries electricity consumption** | **4** |
| **15** | **ELE10** | **Textile industry electricity consumption** | **4** |
| **16** | **ELE11** | **Computer, communications and other electronic equipment manufacturing electricity consumption** | **4** |
| **17** | **ELE12** | **General equipment manufacturing electricity consumption** | **4** |
| **18** | **ELE13** | **Metal products industry electricity consumption** | **4** |
| **19** | **ELE14** | **Non-metallic mineral products industry electricity consumption** | **4** |
| **20** | **HOD** | **Hour of day** | **1** |
| **21** | **O_3_** | **Concentration of O_3_** | **2** |

*Data source:

1.Calculation

2. Department of Ecology and Environment website (<http://sthjt.ah.gov.cn/site/tpl/5371>)

3. NOAA website (<http://www.cdc.noaa.gov>)

4. State Grid Anhui Electric Power Corporation

Table S2

|  | **2018** | | **2019** | | **2020** | |
| --- | --- | --- | --- | --- | --- | --- |
|  | **O_3_(mg/m^3^)** | **Days of pollution** | **O_3_(mg/m^3^)** | **Days of pollution** | **O_3_(mg/m^3^)** | **Days of pollution** |
| **Central Region** | **72.05** | **424** | **68.37** | **376** | **66.84** | **189** |
| Bengbu | 77.09 | 59 | 67.52 | 26 | 70.31 | 20 |
| Chuzhou | 72.88 | 57 | 71.52 | 48 | 70.76 | 30 |
| Fuyang | 69.17 | 36 | 74.74 | 56 | 69.19 | 23 |
| Hefei | 65.09 | 41 | 64.06 | 53 | 57.89 | 17 |
| Huainan | 77.37 | 63 | 73.59 | 55 | 74.30 | 38 |
| Liuan | 78.09 | 45 | 63.44 | 20 | 75.45 | 27 |
| Maanshan | 68.97 | 62 | 67.95 | 62 | 59.37 | 19 |
| Wuhu | 67.74 | 61 | 64.15 | 56 | 57.41 | 15 |
|  |  |  |  |  |  |  |
| **Northern Region** | **77.43** | **197** | **75.94** | **181** | **74.85** | **126** |
| Bozhou | 76.99 | 60 | 76.95 | 59 | 76.46 | 44 |
| Huaibei | 76.83 | 65 | 76.12 | 62 | 75.14 | 45 |
| Suzhou | 78.48 | 72 | 74.75 | 60 | 72.96 | 37 |
|  |  |  |  |  |  |  |
| **Southern Region** | **62.29** | **88** | **66.65** | **118** | **64.02** | **43** |
| Anqing | 74.89 | 37 | 75.70 | 45 | 71.99 | 12 |
| Chizhou | 62.54 | 29 | 66.60 | 45 | 58.05 | 18 |
| Huangshan | 46.44 | 0 | 63.95 | 8 | 62.80 | 1 |
| Tongling | 59.22 | 16 | 61.50 | 16 | 56.84 | 5 |
| Xuancheng | 68.34 | 6 | 65.49 | 4 | 70.44 | 7 |
|  |  |  |  |  |  |  |
| **Total** | **70.01** | **709** | **69.25** | **675** | **67.46** | **358** |

**Figures**


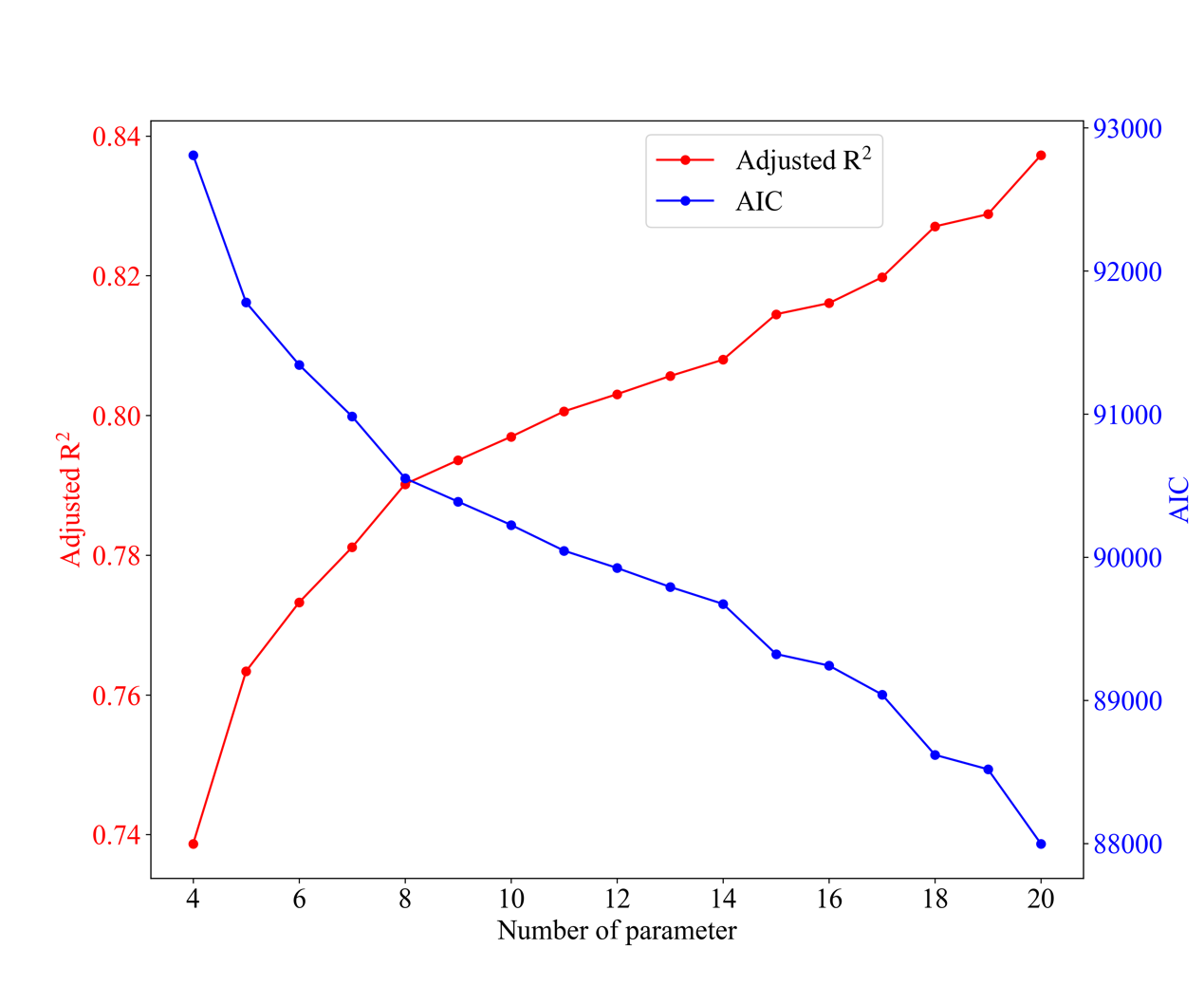


**Fig. S1** AIC and R^2^ of the GAM model with a different number of input variables for Hefei City


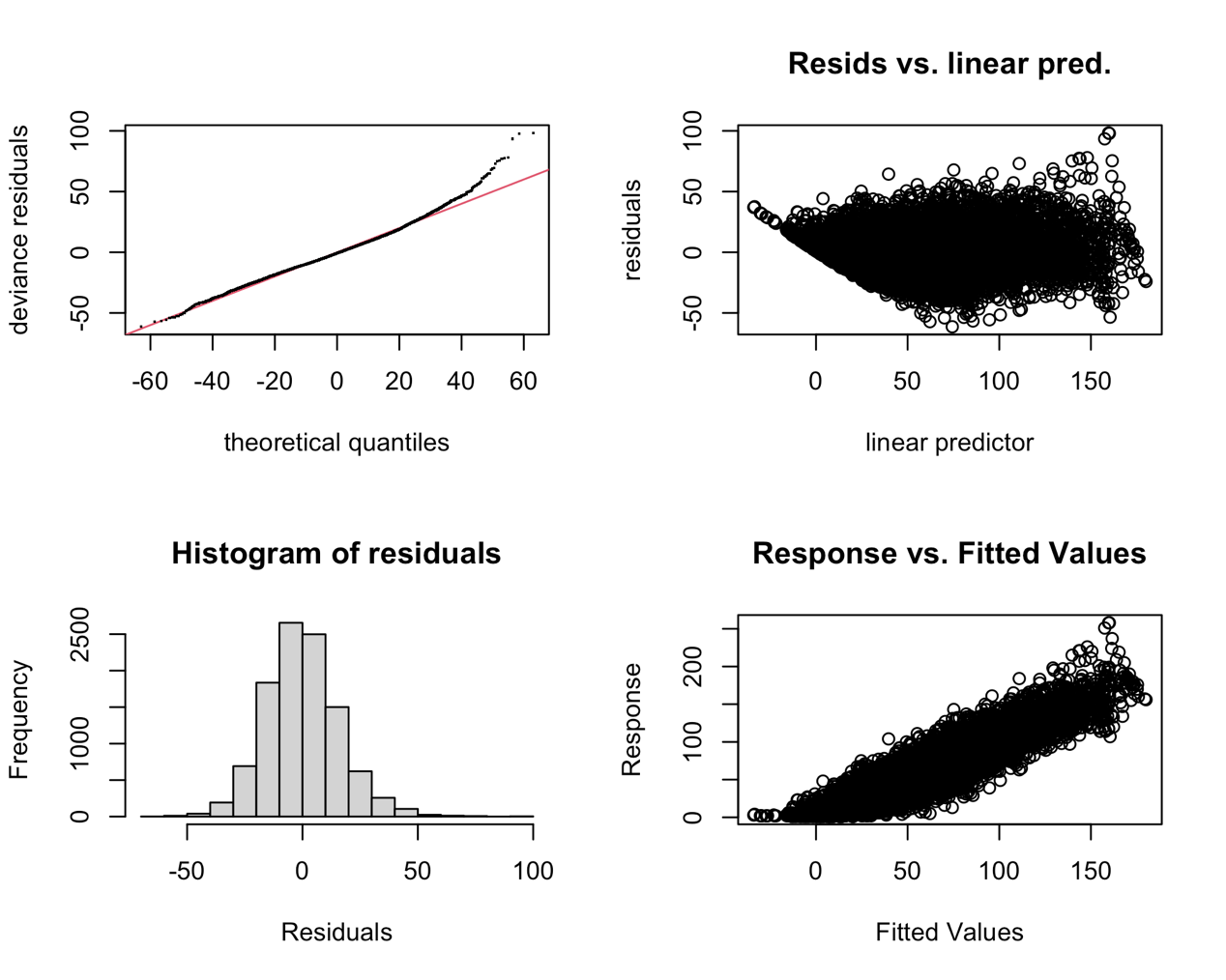


**Fig. S2** The results of the model quality control for Hefei City. The top left is Q-Q plot; Top right is a histogram of the residuals vs the model predicted values; the Bottom left is a histogram of residuals; the Bottom right is the observed O_3_ vs predicted O_3_
